# Involvement of Intercellular Adhesion Molecule-1 Up-Regulation in Bradykinin Promotes Cell Motility in Human Prostate Cancers

**DOI:** 10.3390/ijms140713329

**Published:** 2013-06-26

**Authors:** Hsin-Shan Yu, Tien-Huang Lin, Chih-Hsin Tang

**Affiliations:** 1 Graduate Institute of Basic Medical Science, China Medical University, Taichung 40402, Taiwan; E-Mail: u9682024@cmu.edu.tw; 2 Department of Urology, Buddhist Tzu Chi General Hospital Taichung Branch, Taichung 42743, Taiwan; E-Mail: u9857003@cmu.edu.tw; 3 School of Chinese Medicine, China Medical University, Taichung 40402, Taiwan; 4 Department of Pharmacology, School of Medicine, China Medical University, Taichung 40402, Taiwan; 5 Department of Biotechnology, College of Health Science, Asia University, Taichung 41354, Taiwan

**Keywords:** bradykinin, migration, ICAM-1, prostate cancer

## Abstract

Prostate cancer is the most commonly diagnosed malignancy in men and shows a predilection for metastasis to distant organs. Bradykinin (BK) is an inflammatory mediator and has recently been shown to mediate tumor growth and metastasis. The adhesion molecule intercellular adhesion molecule-1 (ICAM-1) plays a critical role during tumor metastasis. The aim of this study was to examine whether BK promotes prostate cancer cell migration via ICAM-1 expression. The motility of cancer cells was increased following BK treatment. Stimulation of prostate cancer cells with BK induced mRNA and protein expression of ICAM-1. Transfection of cells with ICAM-1 small interfering RNA reduced BK-increased cell migration. Pretreatment of prostate cancer cells with B2 receptor, phosphatidylinositol 3-kinase (PI3K), Akt, and activator protein 1 (AP-1) inhibitors or mutants abolished BK-promoted migration and ICAM-1 expression. In addition, treatment with a B2 receptor, PI3K, or Akt inhibitor also reduced BK-mediated AP-1 activation. Our results indicate that BK enhances the migration of prostate cancer cells by increasing ICAM-1 expression through a signal transduction pathway that involves the B2 receptor, PI3K, Akt, and AP-1. Thus, BK represents a promising new target for treating prostate cancer metastasis.

## 1. Introduction

Prostate cancer is the most commonly diagnosed malignancy in the USA and other western countries [1]. Surgical treatment alone (*i.e.*, radical prostatectomy) often successfully improves the prognoses of patients at early stages of prostate cancer and it is the most frequent therapeutic intervention. As in many cancers, systemic interventions to inhibit the growth and spread of secondary metastasis are necessary for the treatment of advanced patients with disease state [2].

The complex and multi-stage process of formation of secondary tumors at distant sites, called metastasis, is the major cause of mortality in cancer patients [3]. Typically, several steps are involved in the metastasis process, including cellular transformation and tumor growth, angiogenesis, lymphangiogenesis, entry of cancer cells in the circulation by intravasation, invasion of the target organ by extravasation, anchorage and/or attachment to the target organ, and proliferation within the organ parenchyma [4]. The relationship between the migration activity of cancer cells and many of these steps is close and, therefore correlated with tumor metastasis. The inducible surface glycoprotein intercellular adhesion molecule 1 (ICAM-1, also called CD54) belongs to the immunoglobulin supergene family and mediates adhesion-dependent cell-to-cell interactions [5–7]. The extracellular domain of ICAM-1 is crucial for leukocytes and has been shown to be correlated with the transendothelial migration of leukocytes from the capillary bed to the surrounding tissue [8]. ICAM-1 may also promote the migration of other cell types [9–11]. Previous studies have shown that ICAM-1 is involved in breast and lung cancer cell invasion [12,13]. Knockdown of the ICAM-1 expression decreased the invasive activity of breast cancer cells [12]. Thus, ICAM-1 may play a critical role in tumorigenesis, and determining the critical role of ICAM-1 disruption may prevent cancer cell metastasis in disease development.

Bradykinin (BK) will be produced immediately in case of inflammation or injury [14]. It is known that levels of BK may mediate many types of proinflammatory effects, including the contraction of smooth muscles, eicosanoid synthesis, enhanced vascular permeability, vasodilation, and the release of neuropeptides [14,15]. The effects of BK are mediated via 2G-protein-coupled receptors, B1 and B2, which have been pharmacologically characterized and defined by molecular cloning [16,17]. Several recent studies indicated that BK can promote proliferation, invasion, and migration in different cell types via BK receptors, suggesting that BK receptors play critical roles in tumor metastasis [18–20]. Interestingly, the B2 receptor, rather than the B1 receptor, is commonly involved in most biological actions that are BK-mediated [20,21].

Previous studies have reported that BK mediated the invasion and migration of human cancer cells [19,22]. We reported previously that BK promotes migration of human prostate cancer cells through up-regulation of matrix metalloproteinase (MMP)-9 [23]. Here, we further examined whether ICAM-1 expression is involved in BK-mediated motility of human prostate cancer cells. Our results suggest that BK enhanced cell migratory ability and ICAM-1 expression in human prostate cancer cells. Furthermore, BK-mediated cell migratory ability and ICAM-1 expression may be regulated via the B2 receptor, PI3K, Akt, c-Jun, and AP-1 signaling pathways.

## 2. Results

### 2.1. Involvement of ICAM-1 Up-Regulation in Bradykinin Promotes Cell Motility

**Figure 1:**
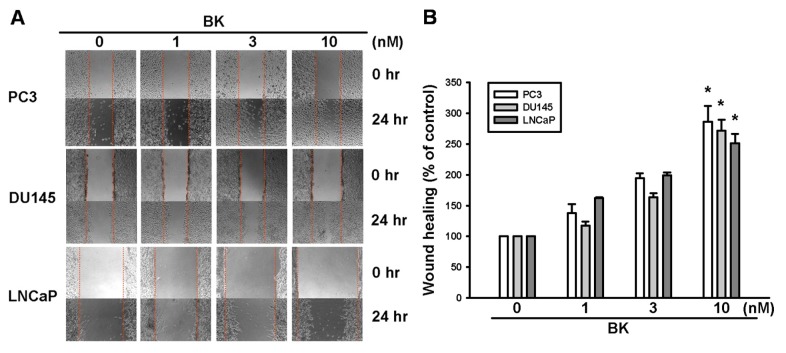

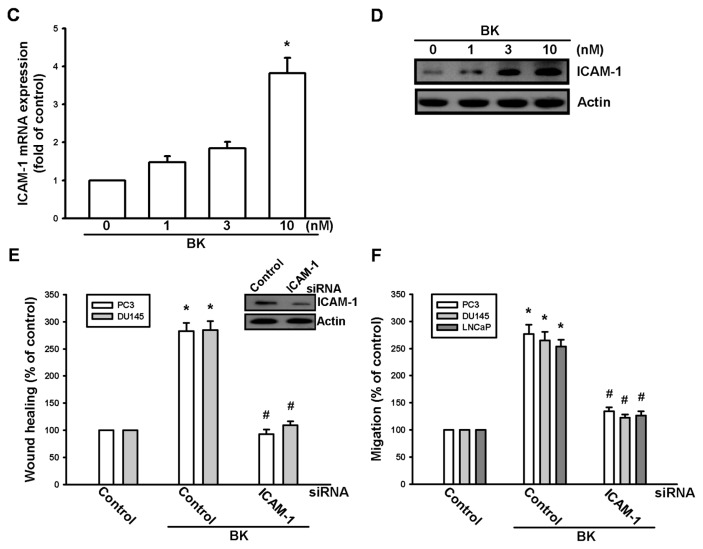
Bradykinin (BK)-directed migration activity of human prostate cancer cells involves up-regulation of intercellular adhesion molecule 1 (ICAM-1). (**A**,**B**) Cells were incubated with various concentrations of BK (1–10 nM). The *in vitro* migration activities were measured by using a wound-healing assay after 24 h. Quantitative data are shown in (**B**); (**C**,**D**) PC3 cells were incubated with BK (10 nM) for 24 h, and the mRNA and protein levels of ICAM-1 were determined using quantitative polymerase chain reaction and Western blotting, respectively; (**E**,**F**) Cells were transfected with ICAM-1 small interfering RNA for 24 h followed by stimulation with BK (10 nM). The *in vitro* migration activity was measured by using a wound-healing assay and Transwell assay. Results are expressed as the mean ± SE. *p < 0.05 compared with control. ^#^*p* < 0.05 compared with BK-treated group.

We previously reported that BK increases cell migration of prostate cancer cells by using a Transwell assay [23]. In this study, the motility of prostate cancer cells was examined by using wound-healing migration and Transwell migration assays. Stimulation of human prostate cancer cells (PC3, DU145, and LNCaP cells) with BK promoted wound-healing migration activity (Figure 1A,B). ICAM-1 activation has been reported to mediate prostate cancers motility [24,25]. Therefore, we hypothesized that ICAM-1 may be also involved in the BK-induced migratory ability of human prostate cancer cells. Incubation of cells with BK increased the mRNA and protein expressions of ICAM-1 dose dependently (Figure 1C,D). To confirm that ICAM-1 plays an important role in BK-induced cell migration, ICAM-1 siRNA was used. Transfection of cells with ICAM-1 siRNA blocked ICAM-1 expression and BK-mediated cell migration, which was demonstrated in wound-healing and Transwell assays (Figure 1E,F). Therefore, these results indicate that ICAM-1 up-regulation is involved in BK-promoted cell migration of human prostate cancer cells.

### 2.2. Involvement of the B2 Receptor in BK-Mediated Migration and ICAM-1 Up-Regulation of Human Prostate Cancer Cells

**Figure 2:**
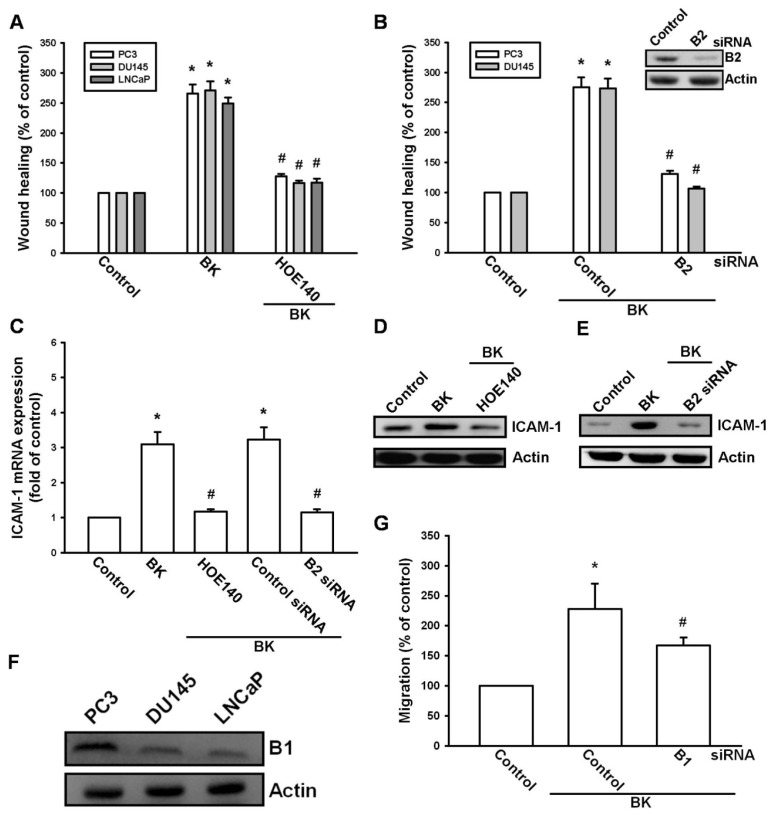
Bradykinin (BK) increased cell migration and intercellular adhesion molecule 1 (ICAM-1) expression through the B2 receptor. (**A**,**B**) Cells were pretreated with B2 receptor antagonist HOE140 (10 nM) for 30 min or transfected with B2 small interfering RNA (siRNA) for 24 h followed by stimulation with BK (10 nM). The *in vitro* migration was measured by using a wound-healing migration assay; (**C**–**E**) PC3 cells were pretreated with the B2 receptor antagonist HOE140 (10 nM) for 30 min or transfected with B2 siRNA for 24 h followed by stimulation with BK (10 nM) for 24. The mRNA and protein levels of ICAM-1 were determined by quantitative polymerase chain reaction (PCR) and Western blotting, respectively; (**F**) Total proteins were extracted from PC3, DU145, and LnCaP cells and subjected to Western blotting for B1 receptor detection; (**G**) PC3 cells were transfected with B1 siRNA for 24 h followed by stimulation with BK (10 nM). The *in vitro* migration was measured by using a Transwell assay. Results are expressed as the mean ± SE. *p < 0.05 compared with control. ^#^*p* < 0.05 compared with BK-treated group.

It has been reported that the expression of B2 is associated with a metastatic phenotype of prostate cancer cell lines [23]. Therefore, we investigated whether the B2 receptor mediates BK-induced prostate cancer cell migration and ICAM-1 expression. Pretreatment of cells with the B2 receptor antagonist HOE140 abolished BK-mediated cell migration (Figure 2A). To further confirm the role of the B2 receptor in BK-mediated motility, B2 specific siRNA was used. The protein level of the B2 receptor and BK-mediated cell migration decreased when cells were transfected with siRNA against the B2 receptor (Figure 2B). Furthermore, pretreatment of cells with HOE140 or transfection with B2 receptor siRNA also reduced the BK-mediated expression of ICAM-1 (Figure 2C–E). Next, we examined the expression of B1 receptor in human prostate cancer cell lines. We found that all three prostate cancer cell lines expressed the B1 receptor (Figure 2F). However, transfection of PC3 cells with B1 siRNA did not affect BK-induced cell migration (Figure 2G), suggesting that B1 is involved in BK-mediated cell motility. Therefore, the BK/B2 interaction is involved in cell migration and ICAM-1 expression in human prostate cancer cells.

### 2.3. PI3K and Akt Signaling Pathways Are Involved in BK-Induced Cell Migration and ICAM-1 Expression

**Figure 3:**
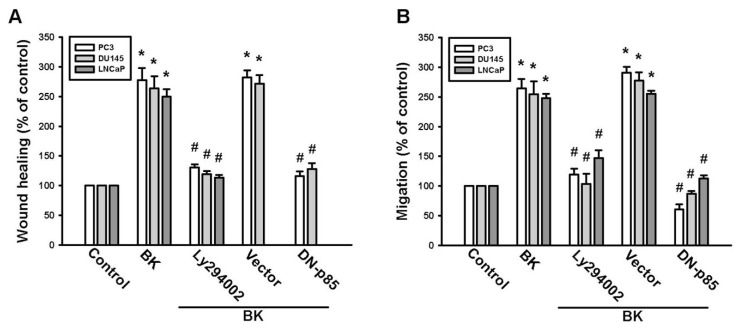

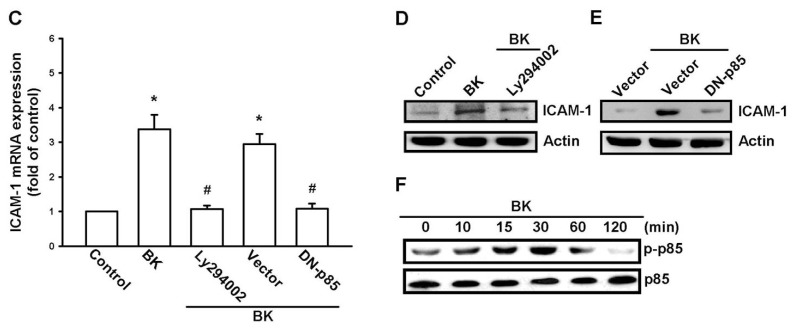
The phosphatidylinositol 3-kinase (PI3K) pathway is involved in bradykinin (BK)-mediated migration and intercellular adhesion molecule 1 (ICAM-1) expression in human prostate cancer cells. (**A**,**B**) Cells were pretreated for 30 min with Ly294002 (3 μM) or transfected with a p85 mutant for 24 h followed by stimulation with BK (10 nM). The *in vitro* migration was measured by using wound-healing and Transwell assays; (**C**–**E**) PC3 cells were pretreated with for 30 min with Ly294002 (3 μM) or transfected with a p85 mutant for 24 h followed by stimulation with BK (10 nM) for 24 h. The mRNA and protein levels of ICAM-1 were examined by quantitative polymerase chain reaction and Western blotting, respectively; (**F**) PC3 cells were incubated with BK (10 nM) for the indicated time intervals, and p85 phosphorylation was examined by Western blotting. Results are expressed as the mean ± SE. *p < 0.05 compared with control. ^#^*p* < 0.05 compared with BK-treated group.

**Figure 4:**
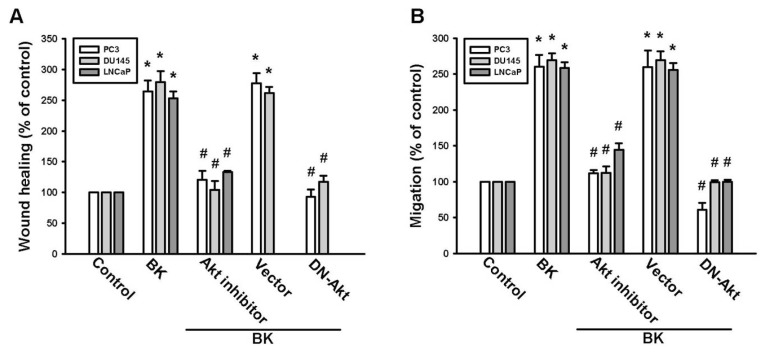

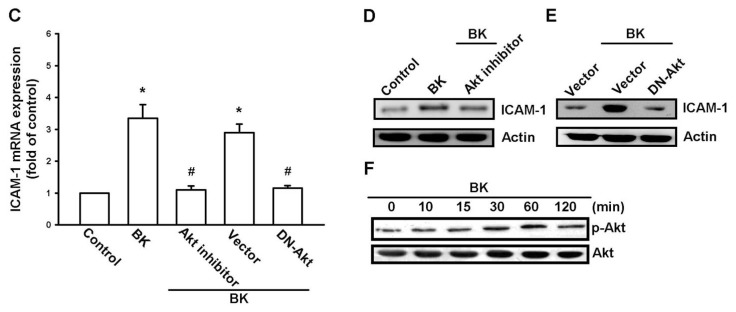
The Akt pathway is involved in BK-mediated migration and intercellular adhesion molecule 1 (ICAM-1) expression in human prostate cancer cells. (**A**,**B**) Cells were pretreated for 30 min with Akt inhibitor (10 μM) or transfected with Akt mutant for 24 h followed by stimulation with BK (10 nM). The *in vitro* migration was measured by using wound-healing and Transwell assays; (**C**–**E**) PC3 cells were pretreated for 30 min with an Akt inhibitor (10 μM) or transfected with an Akt mutant for 24 h followed by stimulation with BK (10 nM). The mRNA and protein levels of ICAM-1 were determined by quantitative polymerase chain reaction and Western blotting, respectively; (**F**) PC3 cells were incubated with BK (10 nM) for the indicated time intervals, and Akt phosphorylation was examined by Western blotting. Results are expressed as the mean ± SE. *p < 0.05 compared with control. ^#^*p* < 0.05 compared with BK-treated group.

PI3K/Akt activation has been reported to occur in BK-mediated cell functions [26,27]. To examine whether PI3K is involved in BK-induced cell migration, the PI3K inhibitor Ly294002 was used. Pretreatment of cells with the PI3K inhibitor Ly294002 blocked BK-mediated migration and ICAM-1 expression (Figure 3A–D). To confirm the role of PI3K in BK-mediated motility, we transfected cells with a p85 mutant, which also reduced BK-increased cell migration and expression of ICAM-1 (Figure 3A–C,E). Direct incubation of cells with BK increased the phosphorylation of p85 (Figure 3F). The PI3K-dependent signaling pathway causes enzymatic activation of Akt via phosphorylation at Ser^473^ residue [28]. We next examined whether PI3K-dependent Akt activation is involved in BK-induced cell migration and ICAM-1 expression. Pretreatment of cells with Akt inhibitor or transfection of cells with Akt mutant antagonized BK-promoted cell migration and ICAM-1 expression (Figure 4A–E). In addition, treatment of PC3 cells with BK promoted Akt Ser^473^ phosphorylation time dependently. Our data suggest that BK increases the expression of ICAM-1 and cell motility through PI3K and Akt signaling pathways in human prostate cancer cells.

### 2.4. AP-1 Is Involved in BK-Mediated Migration and ICAM-1 Expression

**Figure 5:**
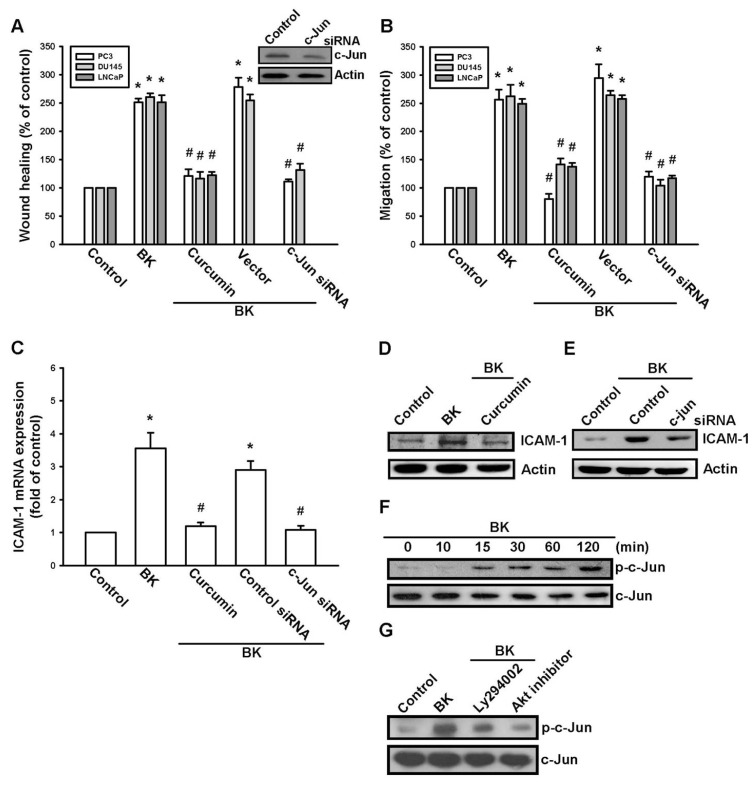
Bradykinin (BK) induces cell migration and intercellular adhesion molecule 1 (ICAM-1) up-regulation through AP-1. (**A**,**B**) Cells were pretreated for 30 min with curcumin (3 μM) or transfected with c-Jun siRNA followed by stimulation with BK (10 nM). The *in vitro* migration was measured by using wound-healing and Transwell assays; (**C**–**E**) PC3 cells were pretreated for 30 min with curcumin (3 μM) or transfected with c-Jun siRNA followed by stimulation with BK (10 nM), the mRNA and protein levels of ICAM-1 were examined by quantitative PCR and Western blotting, respectively; (**F**,**G**) PC3 cells were incubated with BK for the indicated time intervals or pretreated with Ly294002 and Akt inhibitor for 30 min followed by stimulation with BK (10 nM) for 120 min, c-Jun phosphorylation was examined by Western blotting. Results are expressed as the mean ± SE. *p < 0.05 compared with control. ^#^*p* < 0.05 compared with BK-treated group.

A previous study has shown that AP-1 activation mediates ICAM-1 expression and metastasis in human prostate cancer cells [29]. To examine whether AP-1 activation is involved in the signal transduction pathway leading to BK-induced increased ICAM-1 expression and cell motility, the AP-1 inhibitor curcumin [30] was used. Curcumin antagonized the increase in the migration and ICAM-1 expression induced by BK (Figure 5A–D). AP-1 activation was further evaluated by analyzing c-Jun activation. Transfection of cells with c-Jun siRNA reduced the BK-mediated increase in cell migration and ICAM-1 expression (Figure 5A–C,E). Stimulation of cells with BK increased c-Jun phosphorylation (Figure 5F). Furthermore, pretreatment of cells with Ly294002 or an Akt inhibitor reduced the BK-induced c-Jun phosphorylation (Figure 5G). These data indicate that AP-1 is involved in BK-mediated cell migration and expression of ICAM-1 in human prostate cancer cells.

**Figure 6:**
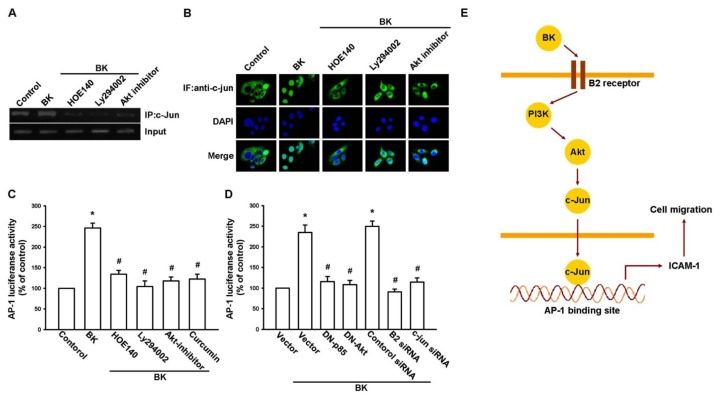
Bradykinin (BK) induced AP-1 activation through the B2 receptor/PI3K/Akt pathway. (**A**) PC3 cells were pretreated with HOE140, Ly294002, or Akt inhibitor for 30 min and then stimulated with BK (10 nM) for 120 min. Next, a chromatin immunoprecipitation assay was performed. Chromatin was immunoprecipitated with a c-Jun antibody. The precipitated chromatin was used as a loading control (input); (**B**) PC3 cells were pretreated with HOE140, Ly294002 or Akt inhibitor for 30 min and then stimulated with BK (10 nM) for 120 min, c-Jun immunofluorescence staining was then observed; (**C**) PC3 cells were pretreated with HOE140, Ly294002, Akt inhibitor, and curcumin for 30 min or (**D**) transfected with a p85 mutant and an Akt mutant and with B2 and c-Jun small interfering RNAs before exposure to BK (10 nM). AP-1 luciferase activity was measured; the results were normalized to β-galactosidase activity and expressed as the mean ± SE of 3 independent experiments performed in triplicate. Results are expressed as the mean ± SE. *p < 0.05 compared with control. ^#^*p* < 0.05 compared with BK-treated group; (**E**) Schematic presentation of the signaling pathways involved in BK-induced cell migration and ICAM-1 expression of human prostate cancer cells. BK activates B2 receptor, PI3K, and Akt pathways, which in turn induce AP-1 activation, which leads to ICAM-1 expression and increases the migration of human prostate cancer cells.

The *in vivo* recruitment of c-Jun to the ICAM-1 promoter was assessed via a chromatin immunoprecipitation assay [31]. *In vivo* binding of c-Jun to the AP-1 element of the ICAM-1 promoter occurred after BK stimulation (Figure 6A). The binding of c-Jun to the AP-1 element in the presence of BK was reduced by HOE140, Ly294002, or an Akt inhibitor (Figure 6A). Stimulation of cells with BK also increased c-Jun translocation to the nucleus (Figure 6B). In addition, pretreatment of cells with HOE140, Ly294002, or an Akt inhibitor decreased BK-induced c-Jun translocation (Figure 6B). Furthermore, BK increased AP-1 luciferase activity, which was used as an indicator of AP-1 activation (Figure 6C). Pretreatment of cells with HOE140, Ly294002, an Akt inhibitor, or curcumin antagonized the BK-mediated AP-1-luciferase activity (Figure 6C). In addition, the BK-induced increase in AP-1-luciferase activity was also abolished by co-transfection of cells with p85 and Akt mutants or B2 and c-Jun siRNAs (Figure 6D). Therefore, the B2 receptor, PI3K, and Akt are upstream molecules for the BK-mediated activation of AP-1 in human prostate cancer cells.

## 3. Discussion

A characteristic of prostate cancer cells is their striking tendency to undergo metastasis [3,32]. A analysis of trophic signals that control metastasis of prostate cancer is crucial for the identification of new molecular targets for anti-metastasis therapies. We previously reported that BK promotes cell migration of human prostate cancer cells through MMP-9 expression [23]. In this study, we further investigated whether ICAM-1 up-regulation is also involved in BK-promoted cell motility of human prostate cancer cells. Here, we reported that BK enhanced cell migration and ICAM-1 expression of human prostate cancer cells. One of the mechanisms underlying BK-mediated migration is activation of the B2 receptor, PI3K, Akt, and AP-1 signaling pathways. It has been reported that MMP-9 cleaves ICAM-1 and participates in tumor cell resistance to natural killer cell-mediated cytotoxicity [33]. In the current study, we did not examine whether MMP-9 also cleaved ICAM-1 in BK-mediated cell motility of prostate cancer cells. Therefore, whether MMP-9 degrades ICAM-1 after BK stimulation is needs further examination.

Recently, BK has been reported to mediate the invasion and migration of human cancer cells [19,22]. Here, we exogenously applied BK to prostate cancer cell lines and observed induced cell motility. Exogenously applied BK is representative of the *in vitro* endocrine effect of BK. Previous studies used BK (3–10 nM) as a pathological condition [20,34]. In the current study, we also applied BK at a concentration of 10 nM to induce ICAM-1 expression and cell motility. Therefore, 10 nM BK represents a pathological condition in prostate cancer metastasis.

ICAM-1 is an important adhesion molecule during the adhesion between endothelial cells and cancer cells which is an essential step in cancer invasion [35]. Several lines of evidence in this study show that ICAM-1 is involved in BK-induced cell migration of human prostate cancer cells. First, BK increased ICAM-1 mRNA and protein expressions. Second, BK-induced cell migration was inhibited by using specific ICAM-1 siRNA. This is not the first proof that ICAM-1 is an important mediator in cancer metastasis. In oral cancer, prostaglandin E2 (PGE2) induced cell motility through ICAM-1 up-regulation [36]. On the other hand, interleukin (IL)-6 enhanced osteosarcoma migration involves ICAM-1 expression [37]. Furthermore, CCN3 also promoted ICAM-1 expression and cell motility of human prostate cancer cells [25]. Collectively, these results suggest that ICAM-1is an important target to treat cancer metastasis.

Two types of BK receptors, B1 and B2 have been defined and cloned [38]. We previously reported that the expression of B2 was associated with a metastatic phenotype of prostate cancer cell lines [23]. In addition, blocking of the B2 receptor abolished BK-induced cell migration [23]. Here, we also confirmed this phenomenon, *i.e.*, a B2 receptor antagonist and siRNA reduced BK-induced increased cell motility in a wound-healing assay. Furthermore, pretreatment of cells with a B2 receptor antagonist or transfection of cells with B2 siRNA also decreased BK-mediated ICAM-1 expression. In the current study, we also found that all three prostate cancer cells used expressed the B1 receptor. However, transfection with B1 siRNA did not affect BK-mediated cell migration. Therefore, the B1 receptor is not involved in BK-induced cell motility. Collectively, the results of our previous study and this study suggest that MMP-9 and ICAM-1 expression are involved in BK-mediated cell migration through the B2 receptor.

PI3K-dependent Akt activation has been shown to play an important role in BK-mediated cellular functions [26,27,39]. We demonstrated that treatment with a PI3K inhibitor and Akt inhibitor inhibited BK-induced cell motility and ICAM-1 expression, suggesting that PI3K/Akt activation is a requisite event in BK-increased cell migration in prostate cancer cells. This was confirmed by the observation that p85 and Akt mutants inhibited the increased migration and ICAM-1 expression in human prostate cancer cells. These results suggest that the PI3K-dependent Akt activation is required for BK-induced expression of ICAM-1 and cell migration. In contrast, we previously reported that BK increased prostate cancer migration through the protein kinase Cδ (PKCδ)/c-Src signaling pathway [23]. In this study, we also provide evidence that PI3K/Akt is involved in BK-increased prostate cancer cell migration. Previous studies have reported that the PKCδ/c-Src pathway interacts with the PI3K/Akt pathway in different cell types [40,41]. However, we did not examine the interaction between the PKCδ/c-Src and PI3K/Akt pathways in prostate cancer cells. Whether PKCδ/c-Src interacts with PI3K/Akt after BK stimulation needs further examination.

There are different binding sites for a number of transcription factors in the human ICAM-1 promoter, including nuclear factor kappa B (NF-κB), AP-1, SP, and CCAAT/enhancer-binding protein binding sites [7]. In this study, we found that AP-1 activation is involved in BK-mediated migratory ability and ICAM-1 expression in prostate cancer cells. The AP-1 sequence binds to many members of transcription factor families including the Jun and Fos families. These nuclear proteins interact with the AP-1 site as Jun homodimers or Jun-Fos heterodimers formed by protein dimerization through their leucine zipper motifs. The results of this study show that BK induced c-Jun phosphorylation. Furthermore, c-Jun siRNA abolished BK-induced cell migration and ICAM-1 expression in prostate cancer cells. In addition, BK also enhanced the binding of c-Jun to the AP-1 element in the ICAM-1 promoter, as shown in the chromatin immunoprecipitation assay. Phosphorylation of c-Jun and binding of c-Jun to the AP-1 element as well as c-Jun translocation to the nucleus were blocked by treatment with HOE140, Ly294002, or Akt inhibitor. These results indicate that BK might act through the B2 receptor, PI3K, Akt, c-Jun, and AP-1 pathways to induce the expression of ICAM-1 and cell migratory ability in human prostate cancer cells. Taken together, our results provide evidence that BK up-regulates migration and ICAM-1 expression of human prostate cancer cells via the B2 receptor/PI3K/Akt signaling pathway.

## 4. Experimental Section

### 4.1. Materials

BK was purchased from Calbiochem (San Diego, CA, USA). Anti-mouse and anti-rabbit IgG-conjugated horseradish peroxidase, rabbit polyclonal antibodies specific forp85, p-p85, Akt, p-Akt (Ser^473^), c-Jun, p-c-Jun, ICAM-1,β-actin, and siRNA against ICAM-1, c-Jun, and control (negative control for experiments using targeted siRNA transfection; each consisted of a scrambled sequence that would not cause specific degradation of any known cellular mRNA) were purchased from Santa Cruz Biotechnology (Santa Cruz, CA, USA). The B2 receptor antagonist HOE140 was purchased from Tocris Bioscience (Ellisville, MO, USA). Ly294002 and Akt inhibitor (1L-6-hydroxymethyl-chiro-inositol-2-((*R*)-2-Omethyl-3-*O*-octadecylcarbonate)) were purchased from Calbiochem. The AP-1 luciferase plasmid was purchased from Stratagene (La Jolla, CA, USA). The p85 (Δp85; deletion of 35 amino acids, *i.e.*, residues 479 513, of p85) and Akt (Akt K179A) dominant-negative mutants were gifts from Dr. W.M. Fu (National Taiwan University, Taipei, Taiwan). The pSV-β-galactosidase vector and luciferase assay kit were purchased from Promega (Madison, MA, USA). All other chemicals were purchased from Sigma–Aldrich (St. Louis, MO, USA).

### 4.2. Cell Culture

Human prostate cancer cell lines (PC3, DU145, and LNCaP) were purchased from the American Type Culture Collection (Manassas, VA, USA). The cells were maintained in RPMI-1640 medium which was supplemented with 20 mM HEPES and 10% heat-inactivated fetal calf serum (FCS), 2 μM glutamine, penicillin (100 U/mL), and streptomycin (100 μg/mL) at 37 °C in 5% CO_2_.

### 4.3. Migration Assay

The migration assay was performed using Transwell membrane filter inserts (Costar, NY, USA; pore size, 8-μm) in 24-well dishes. Before performing the migration assay, cells were pretreated for 30 min with different concentrations of inhibitors, including HOE140, Ly294002, Akt inhibitor, curcumin, or vehicle control (0.1% dimethyl sulfoxide). Approximately 1 × 10^4^ cells in 200 μL of serum-free medium were placed in the upper chamber, and 300 μL of the same medium containing BK were placed in the lower chamber. The plates were incubated for 24 h at 37 °C in 5% CO_2_, and cells were then fixed in 1% formaldehyde for 5 min and stained with 0.05% crystal violet in phosphate-buffered saline (PBS) for 15 min. Cells on the upper side of the filters were removed with cotton-tipped swabs, and the filters were washed with PBS. Cells on the underside of the filters were examined and counted under a microscope. Each treatment was assayed in triplicate in each experiment, and each experiment was repeated at least 3 times [36].

### 4.4. Wound-Healing Migration Assay

For the wound-healing migration assay, cells were seeded on 12-well plates at a density of 1 × 10^5^ cells/well in culture medium. At 24 h after seeding, monolayer cells were manually scratched with a pipette tip to create extended and definite scratches in the center of the dishes with a bright and clear field (~2 mm). The detached cells were removed by washing the dishes once with PBS. Serum-free medium with or without BK was added to each dish as indicated after pretreatment with the inhibitors for 30 min. After 24 h of migration, the numbers of migrated cells were counted from the resulting three images obtained with a light microscope (Nikon TS100; Tokyo, Japan) for each point and then averaged for each experimental condition. The results were compared with those of the control group after 24 h of migration.

### 4.5. Quantitative Real-Time Polymerase Chain Reaction

Total RNA was extracted from prostate cancer cells using a TRIzol kit (MDBio Inc., Taipei, Taiwan). The reverse transcription reaction was performed using 2 μg of total RNA (in 2 μL RNase-free water) that was reverse transcribed into cDNA with an MMLV RT kit (Promega), following the manufacturer’s recommended procedures [42,43]. Quantitative real-time polymerase chain reaction (qPCR) analysis was carried out with the TaqMan one-step PCR Master Mix (Applied Biosystems, Foster City, CA, USA). cDNA template (2 μL) was added to each 25-μL reaction with sequence-specific primers and TaqMan probes. All target gene primers and probes were purchased commercially (β-actin was used an internal control) (Applied Biosystems). qPCR assays were carried out in triplicate on a StepOnePlus sequence detection system. The cycling conditions were as follows: polymerase activation at 95 °C for 10 min, followed by 40 cycles at 95 °C for 15 s and 60 °C for 60 s. The threshold was set above the non-template control background and within the linear phase of the target gene amplification to calculate the cycle number at which the transcript was detected (denoted C_T_).

### 4.6. Western Blot Analysis

Cell lysates were prepared as described previously [44,45]. Proteins were resolved on SDS-PAGE and transferred to Immobilon polyvinyldifluoride (PVDF) membranes. The blots were blocked with 4% bovine serum albumin (BSA) for 1 h at room temperature and then probed with rabbit anti-human antibodies against p85, p-p85, Akt, p-Akt, c-Jun, p-c-Jun, ICAM-1, or β-actin (1:1000) for 1 h at room temperature. After three washes, the blots were subsequently incubated with a donkey anti-rabbit peroxidase-conjugated secondary antibody (1:1000) for 1 h at room temperature. The blots were visualized with enhanced chemiluminescence using Kodak X-OMAT LS films (Eastman Kodak, Rochester, NY, USA).

### 4.7. Reporter Gene Assay

Prostate cancer cells were transfected with reporter plasmids using Lipofectamine 2000 according to the manufacturer’s recommendations. Twenty-four hours after transfection, the cells were treated with inhibitors for 30 min and BK or vehicle was then added for 24 h. Cell extracts were then prepared, and luciferase and β-galactosidase activities were measured [36].

### 4.8. Chromatin Immunoprecipitation Assay

Chromatin immunoprecipitation (ChIP) analysis was performed as described previously [22]. DNA immunoprecipitated by using a anti-c-Jun monoclonal antibody (mAb) was purified. The DNA was then extracted with phenol–chloroform. The purified DNA pellet was subjected to PCR. PCR products were then resolved by 1.5% agarose gel electrophoresis and visualized by UV transillumination. The primers 5′-AGACCTTAGCGCGGTGTAGA-3′ and 5′-AGTAGCAGAGGAGCTCAGCG-3′ were utilized to amplify across the ICAM-1 promoter region (-346 to -24) [31].

### 4.9. Immunofluorescence Staining

Cells were cultured in 12-mm coverslips. After treatment with BK, cells were fixed with 4% paraformaldehyde at room temperature. Thirty minutes later, 4% nonfat milk in PBS containing 0.5% Triton X-100 was added to the cells. The cells were then incubated with mouse anti-c-Jun (1:100) and fluorescein isothiocyanate (FITC)-conjugated goat anti-rabbit secondary antibody (1:500; Leinco Technology Inc., St. Louis, MO, USA) for 1 h, respectively. The fluorescein isothiocyanate-positive cells (FITC) were detected using a Zeiss fluorescence microscope.

### 4.10. Statistics

The values given represent the means ± SEM. The significances of difference between the experimental groups and controls was assessed by using the Student’s *t* test. Difference were significant if the *p* value was <0.05.

## 5. Conclusions

Here, we present a new mechanism for the BK-mediated migratory ability of prostate cancer cells, *i.e.*, by up-regulation of ICAM-1. BK increased the expression of ICAM-1 through the B2 receptor, PI3K, Akt, and AP-1-signaling pathway and migration of human prostate cancer cells (Figure 6E). In addition, the discovery of the BK-mediated signaling pathway may lead us to understanding the mechanism of human prostate cancer metastasis and to help us to develop effective therapies in the future.
